# Extracellular RNA in iron-induced hepatic injury: Beyond TLR3 toward an integrated inflammatory axis

**DOI:** 10.1016/j.toxrep.2025.102126

**Published:** 2025-09-11

**Authors:** Hector A. Cabrera-Fuentes, Klaus T. Preissner

**Affiliations:** a División de Estudios de Posgrado e Investigación, Tecnológico Nacional de México / Instituto Tecnológico de Tijuana, Tijuana, B.C 22414, Mexico; b R&D group, Vice Presidency Scientific Research & Innovation, Imam Abdulrahman bin Faisal University (IAU), P.O. Box 1982, Dammam 31441, Saudi Arabia; c UNAM-UABJO Research Centre, Faculty of Medicine and Surgery, Universidad Autónoma Benito Juárez de Oaxaca (UABJO), Oaxaca 68000, Mexico; d Dirección de la División deInvestigación y Desarrollo Científico, Benemérita Universidad de Oaxaca, Oaxaca 68000, México; e Kerckhoff-Heart ResearchInstitute, Department of Cardiology, Medical School, Justus-Liebig-University, Giessen, Germany

Dear editor,

We read with interest the recent article by Raza et al. [1], proposing that extracellular RNA (eRNA) mediates iron-induced hepatocyte toxicity through TLR3 activation. While the study highlights an important concept linking iron overload to sterile inflammation, several methodological and conceptual limitations restrict the validity of their conclusions. First, the model is restricted to HepG2 cells exposed to supraphysiological FeCl₃ (0.6 mM, 24 h), conditions prone to non-specific oxidative stress rather than pathophysiologic iron overload. Moreover, HepG2 cells, as immortalized hepatocellular carcinoma–derived lines, differ substantially from primary hepatocytes in metabolic capacity, RNA turnover, and innate immune receptor expression. This raises concern that the results may not fully capture the *in vivo* hepatic response. Second, TLR3 attribution rests solely on RNase1 and a pharmacologic inhibitor—both indirect and potentially off-target—without receptor genetics or gain-of-function (purified eRNA transfer). Third, the RNA released under iron stress was not profiled; distinct classes (rRNA, miRNA, lncRNA) can engage different pathways. Reporting of sample sizes/replicates and quantitative densitometry is limited.

Beyond internal constraints, a TLR3-exclusive model conflicts with the literature. In macrophages, eRNA synergizes powerfully with TLR2 ligands to augment cytokines (shifting dose–responses), but not with TLR3 [2], [3]. eRNA alone can polarize macrophages toward an M1 phenotype independently of TLRs [4]. In the same study, genome-wide microarray profiling revealed that eRNA triggers a broad pro-inflammatory gene program—including TNF, IL6, IL1A, IL23A, CCL3/4/20, and CXCL1/6—underscoring its function as a potent endogenous danger signal. Most critically for the ongoing debate, our earlier study on atherosclerosis demonstrated that eRNA accumulates in plaques, activates macrophages, and accelerates lesion growth—effects reversed by RNase1 [5]. When Chen et al. questioned TLR involvement, our response showed that robust eRNA-induced cytokine release could not be reproduced by classical TLR agonists and may instead involve lectin recognition rather than TLR3 [6], [7], [8]. Consistently, Chen et al. later reported that although TLR3 deficiency reduced apoptosis after myocardial ischemia/reperfusion, *in vivo* cytokine production was unaffected, underscoring that eRNA-driven inflammation is largely TLR3-independent [9].

Finally, beyond receptor engagement, eRNA mobilizes cytokines through activation of the TNF-α–converting enzyme (TACE/ADAM17), a pathway our group has characterized extensively. eRNA induces TACE-dependent shedding of TNF-α, thereby amplifying inflammatory loops in vascular injury [10], tumor progression [11], and myocardial ischemia–reperfusion [6]. In our study [6], we delineated a vicious cycle in which TNF-α promotes further eRNA release, escalating oxidative injury, mitochondrial dysfunction, and cell death—an effect that was effectively interrupted by RNase1 [8]. Notably, iron overload itself promotes macrophage M1 polarization and intracellular Fe(II) accumulation, tightly correlated with increased release of TNF-α, IL-1β, and IL-6, as demonstrated by Ma et al. [7]. Thus, in iron-induced hepatic injury, it is equally plausible that Fe(II) accumulation amplifies macrophage activation and TNF-α production through ROS, inflammasome, and eRNA–TACE signaling, rather than exclusively via TLR3.

Taken together, the data presented by Raza et al. [1] suggest an important role of eRNA in iron-induced hepatic inflammation, but they do not provide sufficient evidence to establish a direct eRNA–TLR3 interaction. The lack of RNA species characterization, absence of receptor genetics, and omission of ADAM17/TACE activity or soluble TNF-α measurements leave the mechanism unresolved. In light of existing evidence from cardiovascular, tumor, and now macrophage iron-loading models [2], [3], [6], [7], future work should integrate (i) profiling of released RNA species, (ii) genetic dissection of TLR2/TLR3 pathways, and (iii) direct interrogation of the eRNA–TACE–TNF axis, including ADAM17 activation, pro-TNF cleavage, and RNase1 rescue. Only such an integrated approach will clarify whether eRNA propagates iron-induced sterile inflammation primarily through pattern recognition receptors (PRRs) such as TLR3, or via TACE-dependent cytokine mobilization. Until then, claims of direct eRNA–TLR3 engagement in hepatocyte iron overload remain premature.

## CRediT authorship contribution statement

**Cabrera-Fuentes Hector Alejandro:** Conceptualization, Investigation, Validation, Writing – original draft, Writing – review & editing. **Klaus T. Preissner:** Conceptualization, Investigation, Methodology, Supervision, Validation, Writing – original draft, Writing – review & editing.

## Declaration of Competing Interest

The authors declare that they have no known competing financial interests or personal relationships that could have appeared to influence the work reported in this paper.

## Data Availability

No data was used for the research described in the article.
